# Concurrent advanced HIV disease and viral load suppression in a high-burden setting: Findings from the 2015–6 ZIMPHIA survey

**DOI:** 10.1371/journal.pone.0230205

**Published:** 2020-06-25

**Authors:** S. Balachandra, J. H. Rogers, L. Ruangtragool, E. Radin, G. Musuka, I. Oboho, H. Paulin, B. Parekh, S. Birhanu, K. C. Takarinda, A. Hakim, T. Apollo

**Affiliations:** 1 Division of Global HIV and Tuberculosis, U.S. Centers for Disease Control and Prevention, Bluffhill, Harare, Zimbabwe; 2 PHI/CDC Global Epidemiology Fellowship, U.S. Centers for Disease Control and Prevention, Bluffhill, Harare, Zimbabwe; 3 ICAP New York, New York, NY, United States of America; 4 ICAP Zimbabwe, Avondale, Harare, Zimbabwe; 5 Division of Global HIV and Tuberculosis, U.S. Centers for Disease Control and Prevention, Atlanta, GA, United States of America; 6 Ministry of Health and Child Care, Harare, Zimbabwe; Albert Einstein College of Medicine, UNITED STATES

## Abstract

**Background:**

As Zimbabwe approaches epidemic control of HIV, programs now prioritize viral load over CD4 monitoring, making it difficult to identify persons living with HIV (PLHIV) suffering from advanced disease (AD). We present an analysis of cross-sectional ZIMPHIA data, highlighting PLHIV with AD and concurrent viral load suppression (VLS).

**Methods:**

ZIMPHIA collected blood specimens for HIV testing from 22,501 consenting adults (ages 15 years and older); 3,466 PLHIV had CD4 and VL results. Household HIV testing used the national serial algorithm, and those testing positive then received point-of-care CD4 enumeration with subsequent VL testing. We used logistic regression analysis to explore factors associated with concurrent AD and VLS (<1000 copies/mL). All analyses were weighted to account for complex survey design.

**Results:**

Of the 3,466 PLHIV in the survey with CD4 and VL results, 17% were found to have AD (CD4<200cells/mm^3^). Of all AD patients, 30% had VLS. Concurrent AD and VLS was associated with male sex (aOR 2.45 95%CI 1.61–3.72), older age (35–49 years [aOR 2.46 95%CI 1.03–5.91] and 50+ years [aOR 4.82 95%CI 2.02–11.46] vs 15–24 years), and ART duration (<6 months [aOR 0.46 95%CI 0.29–0.76] and 6–24 months [aOR 2.07 95%CI 1.35–3.17] vs more than 2 years). The relationship between sex and AD is driven by age with significant associations among men aged 25–34, (aOR 3.37 95%CI 1.35–8.41), 35–49 (aOR 5.13 95%CI 2.16–12.18), and 50+ (aOR 12.56 95%CI 4.82–32.72) versus men aged 15–24.

**Conclusions:**

The percentage of PLHIV with AD and VLS illustrates the conundrum of decreased support for CD4 monitoring, as these patients may not receive appropriate clinical services for advanced HIV disease. In high-prevalence settings such as Zimbabwe, CD4 monitoring support warrants further consideration to differentiate care appropriately for the most vulnerable PLHIV. Males may need to be prioritized, given their over-representation in this sub-population.

## Introduction

The HIV epidemic in Zimbabwe has changed dramatically over the past decade. With widespread availability of anti-retroviral therapy (ART), the Ministry of Health and Child Care has implemented critical policies including Option B+ (2014–5), which introduced lifelong ART for all HIV-positive pregnant women, and Treat All (2016–7), eliminating immunologic criteria for ART initiation: these policies have resulted in increased ART coverage and decreased HIV incidence nationwide. As of the end of 2017, there were approximately 1.3 million people living with HIV (PLHIV) in Zimbabwe, and prevalence among 15-49-year olds was estimated at 13.3% (95% confidence Interval (CI) 11.4 to 14.9) [1]. In 2017, the HIV incidence among adults aged 15–49 decreased to 0.54%, representing a 44% reduction in new infections since 2010; this was coincident with an increase in ART coverage from 30% to approximately 84% over that period of time [1]. In 2002, the life expectancy for adults in Zimbabwe was as low as 44 years of age, but by 2016, it had risen to 61 years [2]. Thus, the nation is recovering from what was one of the most crippling epidemics in the world.

Although ART coverage is increasing in sub-Saharan Africa, several recent studies have noted the persistence of advanced disease (AD), defined as CD4 <200 cells/mm^3^ or World Health Organization (WHO) Stage III or IV for adults, adolescents and children >5, among patients initiating ART [3–5]. PLHIV may present with AD due to a delay in HIV diagnosis, ART initiation, disengagement from ART care, or treatment failure, leading to high rates of morbidity and mortality from opportunistic infections (OIs) including tuberculosis (TB) and cryptococcal meningitis [6,7]. As an important tool for treatment prioritization and identification of individuals at significant risk for OIs, WHO continues to recommend baseline CD4 testing at the time of ART initiation. In 2015, they released updated guidelines on the treatment of HIV to emphasize annual viral load (VL) rather than semiannual CD4 monitoring [8]. As attention has shifted towards expanding access to VL monitoring, donor support for CD4 testing has decreased significantly [9]. In its 2014 Country Operational Plan for Zimbabwe, the U.S. President’s Emergency Plan for AIDS Relief (PEPFAR) program contributed $740,000 towards CD4 testing, whereas in subsequent years, CD4 testing has not been part of the PEPFAR portfolio [10]. VL monitoring in Zimbabwe has accelerated rapidly from ~4% coverage nationwide at the end of 2015 to over 30% coverage by the end of 2017; it is difficult to ascertain CD4 coverage among ART patients given the de-prioritization of CD4 testing [11]. As part of a multi-country review of AD from 2004–2015, CD4 results (baseline or otherwise) were noted in 53% of ART patient records across Zimbabwe, though it is fair to assume that this figure has declined over the past several years for the aforementioned reasons [12]. Given the lack of systematically collected CD4 data, the prevalence of AD among PLHIV on ART is unknown.

The Zimbabwe Population-Based HIV Impact Assessment (ZIMPHIA), carried out in 2015–2016, produced nationally and provincially-representative estimates describing PLHIV, including biomarker data on CD4 and VL status, and self-reported data on ART status and TB symptoms (among other factors). In this paper, we present a profile of the sub-population of Zimbabwe’s PLHIV with AD by laboratory marker (CD4 <200), and in particular, the relationship between AD, VL status and duration on ART. Given reports that health-seeking behavior and outcomes among certain populations in Zimbabwe may be a function of their religious affiliation [13,14], we also explored this in relation to the AD population.

## Methods

ZIMPHIA 2015–2016 was a nationally representative, cross-sectional population-based survey of households across Zimbabwe. Stage one of the two-stage stratified cluster sample design selected 499 enumeration areas from the Zimbabwe Population Census 2012 using a probability proportional to size method. Stage two randomly selected a sample of households in each cluster using an equal probability method with an average of 30 households per cluster (range: 15–60). Complete survey methods for the Zimbabwe Population-based HIV Impact Assessment are available online via the ICAP and Columbia University webpage [15].

### Consent and individual interview

Prior to administering individual interviews, written informed consent was obtained via mobile data collection tools from individuals aged 16 years and above and emancipated minors aged 15 years who slept in the household the night before the survey or were usual household members. After parents or guardians of non-emancipated 15-year-olds provided electronically documented permission to approach the minor, written assent was obtained from the 15-year-olds to participate in the interview. Participants provided written consent or assent for participation in the biomarker component of the survey after completing the interview. All consent, permission, and assent procedures and the individual interviews were conducted in either English, Shona, or Ndebele; individuals unable to speak one of these three languages were ineligible to participate.

### Biomarker testing

ZIMPHIA collected whole blood specimens for HIV testing from 22,501 consenting adults (ages 15 years and older), consenting emancipated minors (age 15 years), and assenting unemancipated minors (age 15 years). Household HIV testing used the national serial algorithm of rapid point-of-care tests: Determine (Abbott), First Response (Premier Medical Corporation), and Stat-Pak (Chembio), as a tiebreaker; HIV+ results were confirmed at a satellite laboratory using Geenius, a rapid immunochromatographic assay (Bio-Rad Laboratory, Hercules, California). Participants testing positive for HIV then received point-of-care (PIMA, Abbott, Abbott Park, Illinois) CD4 enumeration using whole blood, with subsequent VL testing in a central laboratory. VL plasma testing was conducted using the Roche (Pleasanton, CA, USA) COBAS^®^ AmpliPrep/COBAS^®^ TaqMan^®^ (CAP/CTM) HIV-1 Test, version 2.0 on a Roche CAP/CTM with 48 analyzer. VL dried blood spot (DBS) testing was done by NucliSENSTM EasyQ HIV-1 v2.0 assay on the bioMérieux (Marcy-l’Étoile, France) NucliSENSTM EasyMAG/EasyQ platform.

### Ethical review

The study protocol and all data collection tools were approved by the Medical Research Council of Zimbabwe (A/1914) and the Institutional Review Boards at the U.S. Centers for Disease Control and Prevention (CDC) (IRB#6702), Westat (IRB# 6317), and Columbia University (IRB# Y06M00).

### Data analysis

We compared the demographic and clinical characteristics of individuals with CD4<200 and ≥200 (cells/ mm^3^) using chi-square tests. We then used logistic regression to identify demographic (age, sex, province, wealth, education, and urban/rural) and clinical (awareness of HIV status, time on ART, history of CD4 test, and VL suppression (VLS)) factors independently associated with AD (CD4 <200 cell/μL). Time on ART was assumed to be < 6 months for all newly-diagnosed participants. In a setting where PLHIV have access to VL but not CD4 testing, patients with both AD and VLS (defined as <1000 copies/ml) may be difficult to identify; we therefore examined factors associated with this combination of traits. All analyses were weighted to account for complex survey design. The multivariable models were developed by first examining the univariate relationship between each of these independent variables and the dependent variables. Each independent variable was added manually to an empty model containing only the dependent variable of interest to conduct a chi-square test, with the appropriate degrees of freedom, on the reduction of the -2 log likelihoods to determine if the independent variable significantly reduced the model deviance. Variables that significantly reduced the model deviance at the p<0.05 level were included in the multivariable logistic regression model to calculate adjusted odds ratios and their 95% CIs. Age was also forced into the multiple variable model as an a priori identified variable of interest.

## Results

### Descriptive statistics of those with advanced disease (CD4 <200) compared to those without advanced disease (CD4 ≥200)

Characteristics of the HIV-positive study population can be found in Table 1. Of the total adult PLHIV in the survey with CD4 and VL results available (3,466), 542 (17%) were found to have advanced disease, with a CD4 <200; 2,924 (83%) had a CD4 ≥200 (Table 1). Among those with AD, 60% were male as compared to 37% in the population with CD4≥200 (p<0.001). Age distribution, residence (urban vs rural), provincial distribution, wealth, and education were similar between the two groups. Of note, there were marked differences between those with CD4<200 and CD4≥200 in the rates of VLS, time since ART initiation, certain religious affiliations, and CD4 test history.

**Table 1:** Characteristics of ZIMPHIA 2015–16 HIV-positive study population disaggregated by CD4 count.

		CD4 <200 cells/mm^3^			CD4> = 200 cells/mm^3^		
** **	**n**	**weighted%**	**95% CI**	**n**	**weighted%**	**95% CI**	**p-value**
**Sex**	**542**	**100.0**		**2,924**	**100.0**		**p<0.001 **
Female	252	39.5	(34.5–44.4)	2,003	63.0	(61.2–64.8)	
Male	290	60.5	(55.6–65.5)	921	37.0	(35.2–38.8)	
**Age**	**542**	**100.0**		**2,924**	**100.0**		**p = 0.11 **
15–24	41	8.9	(6.0–11.9)	298	11.5	(10.0–13.1)	
25–34	117	23.8	(19.5–28.1)	725	27.2	(25.5–28.9)	
35–49	255	47.1	(42.2–52.1)	1,269	43.5	(41.6–45.4)	
50+	129	20.1	(16.1–24.1)	632	17.7	(16.3–19.1)	
**Residence**	**542**	**100.0**		**2,924**	**100.0**		**p = 0.78 **
Rural	377	62.7	(57.5–68.0)	2,019	63.5	(60.8–66.2)	
Urban	165	37.3	(32.0–42.5)	905	36.5	(33.8–39.2)	
**Province**	**542**	**100.0**		**2,924**	**100.0**		**p = 0.14 **
Bulawayo	61	7.8	(5.5–10.2)	336	7.8	(6.8–8.8)	
Manicaland	47	10.0	(7.1–13.0)	236	9.8	(8.2–11.4)	
Mashonaland Central	64	10.3	(7.8–12.7)	248	7.6	(6.1–9.1)	
Mashonaland East	57	11.6	(8.4–14.8)	247	9.9	(7.9–11.8)	
Mashonaland West	44	9.2	(6.0–12.4)	291	11.7	(9.4–14.0)	
Matebeleland North	50	5.0	(3.3–6.8)	382	7.4	(6.5–8.4)	
Matebeleland South	55	6.4	(4.3–8.5)	348	8.1	(6.8–9.4)	
Midlands	47	9.6	(6.4–12.8)	263	10.6	(8.7–12.4)	
Masvingo	60	10.5	(7.3–13.7)	302	10.5	(8.6–12.3)	
Harare	57	19.5	(14.6–24.4)	271	16.7	(14.5–18.8)	
**Wealth Quintile**	**542**	**100.0**		**2,924**	**100.0**		**p = 0.79 **
Lowest	145	21.8	(17.9–25.7)	726	20.4	(18.1–22.7)	
Second	97	16.8	(13.4–20.3)	604	19.0	(17.1–21.0)	
Third	105	18.8	(14.9–22.7)	536	19.1	(16.8–21.4)	
Fourth	102	23.2	(17.5–28.9)	535	22.0	(19.3–24.7)	
Highest	93	19.4	(15.1–23.6)	523	19.4	(17.0–21.9)	
**Education**	**542**	**100.0**		**2,924**	**100.0**		**p = 0.81 **
Primary or lower	231	37.6	(33.0–42.1)	1,245	38.1	(35.8–40.5)	
Secondary or above	311	61.9	(57.9–67.0)	1,679	62.4	(59.5–64.2)	
Virally Suppressed*	**542**	**100.0**		**2,924**	**100.0**		**p<0.001 **
No	375	69.9	(65.2–74.6)	902	34.4	(32.1–36.6)	
Yes	167	30.1	(25.4–34.8)	2,022	65.6	(63.4–67.9)	
**CD4 Test Ever**	**542**	**100.0**		**2,924**	**100.0**		**p<0.001 **
No	252	47.4	(42.2–52.6)	950	35.4	(33.2–37.5)	
Yes	290	52.6	(47.5–57.8)	1,974	64.6	(62.5–66.8)	
**Time Since ART Initiation**	**542**	**100.0**		**2,923**	**100.0**		**p<0.001 **
<6 mos	270	50.6	(45.8–55.4)	982	37.3	(35.1–39.5)	
6 mos to 2 yrs	77	14.6	(11.5–17.8)	447	14.7	(13.2–16.1)	
More than 2 yrs	195	34.8	(30.2–39.4)	1,495	48.1	(45.9–50.2)	
**Religion**	**542**	**100.0**		**2,924**	**100.0**		**p<0.001**
Non-Apostolic Christian	259	46.5	(41.3–51.7)	1501	50.6	(48.2–53.1)	
Apostolic	154	28.2	(23.7–32.8)	960	32.6	(30.1–35.0)	
Traditional	16	2.6	(1.3–4.0)	58	1.9	(1.3–2.5)	
Muslim	5	1.07	(0.07–2.1)	13	0.6	(0.2–0.9)	
Other/None	108	21.6	(17.1–26.0)	392	14.3	(12.8–15.9)	

*viral suppression defined as <1000copies/ml.

### Characteristics associated with advanced disease (CD4 <200)

In bivariate analysis, males were more than twice as likely to have AD as females (crude odds ratio [cOR] 2.61 95% CI 2.07 to 3.29) (Table 2). Additionally, having never received a CD4 test (cOR 1.65 95% CI 1.33 to 2.05) and either initiating ART in the last 6 months (cOR 1.87 95% CI 1.50 to 2.34) or between 6 and 24 months prior to the survey (cOR 1.38 95% CI 1.01 to 1.87) was associated with an increased likelihood of CD4 <200. Identifying as either having no or other religion was associated with increased odds of CD4<200 versus non-Apostolic Christians (cOR 1.64 95% CI 1.23 to 2.18). Not being virally suppressed was also associated with increased odds of advanced HIV disease (cOR 4.45 95% CI 3.49–5.67). Urban vs. rural residence, province, wealth quintile, and education level were not associated with AD, and these variables were excluded from the multivariable logistic regression. Age was included into the multivariable model because there was a trend towards an association between age >35 years compared with the reference group (age 15–24 years) and having a CD4<200. A final, adjusted model with sex, age, CD4 testing history, VLS, time on ART, and religion was calculated. In multivariable analysis, male sex (adjusted odds ratio [aOR] 2.26 95% CI 1.73 to 2.94) and lack of viral suppression (aOR 7.74 95% CI 5.41 to 11.09) maintained associations with AD. A significant interaction was noted between time on ART and VLS which qualitatively changed the direction of the association with AD in the <6 months ART duration group (<6 months aOR 0.54 95% CI 0.34 to 0.87; 6–24 months aOR 1.47 95% CI 1.06 to 2.05). Religious affiliation and a history of not receiving a CD4 test were no longer associated with AD after controlling for the other variables in the model.

**Table 2:** Results of bivariate and multivariable models for advanced HIV disease (CD4 <200 cells/mm^3^ (n = 542)).

	n	cOR (95% CI)*	aOR (95% CI)**
**Sex**			
Female	252	Ref	Ref.
Male	290	2.61 (2.07–3.29)	2.26 (1.73–2.94)
**Age**			
15–24	41	Ref	Ref.
25–34	117	1.13 (0.71–1.79)	1.13 (0.70–1.83)
35–49	255	1.40 (0.93–2.09)	1.63 (1.05–2.52)
50+	129	1.47 (0.95–2.26)	2.09 (1.31–3.32)
**Residence**			
Urban	377	Ref	--
Rural	165	1.03 (0.82–1.30)	--
**Province**			
Bulawayo	61	Ref	--
Manicaland	47	1.02 (0.65–1.61)	--
Mashonaland Central	64	1.35 (0.89–2.05)	--
Mashonaland East	57	1.17 (0.72–1.90)	--
Mashonaland West	44	0.79 (0.51–1.21)	--
Matebeleland North	50	0.68 (0.40–1.14)	--
Matebeleland South	55	0.78 (0.48–1.28)	--
Midlands	47	0.91 (0.58–1.43)	--
Masvingo	60	1.00 (0.64–1.56)	--
Harare	57	1.17 (0.76–1.79)	--
**Wealth Quintile**			
Lowest	145	Ref	--
Second	97	0.83 (0.62–1.10)	--
Third	105	0.92 (0.69–1.23)	--
Fourth	102	0.99 (0.71–1.38)	--
Highest	93	0.93 (0.68–1.28)	--
**Education**			
Primary or below	231	0.98 (0.80–1.20)	--
Secondary or above	311	Ref	--
**Viral Load Suppression**			
Yes	167	Ref	Ref
No	375	4.45 (3.49–5.67)	7.74 (5.41–11.09)
**CD4 test ever**			
No	252	1.65 (1.33–2.05)	0.98 (0.67–1.44)
Yes	290	Ref	Ref.
**Time since ART initiation**			
<6 months	270	1.87 (1.50–2.34)	0.54 (0.34–0.87)
6 months to 2 years	77	1.38 (1.01–1.87)	1.47 (1.06–2.05)
More than 2 years	195	Ref.	Ref.
**Religion**			
Non Apostolic Christian	259	Ref.	Ref.
Apostolic	154	0.94 (0.74–1.21)	0.92 (0.71–1.20)
Traditional	16	1.50 (0.83–2.70)	0.92 (0.48–1.78)
Muslim	5	2.08 (0.73–5.92)	2.15 (0.57–8.13)
Other or None	108	1.64 (1.23–2.18)	1.16 (0.84–1.60)

*cOR (crude Odds Ratio)

**aOR (adjusted Odds Ratio).

### Characteristics associated with advanced disease (CD4 <200) among those with viral load suppression

Seventeen percent of PLHIV were found to have AD. Of these, nearly one-third (30%) would not be identified with VL testing alone because they are virally suppressed. Upon further examination of the sub-population of patients with both AD and VLS (Table 3), bivariate analysis revealed associations with male sex (cOR 2.70 95% CI 1.73 to 4.20), age over 50 years vs 15–24 years (cOR 4.16 95%CI 1.51 to 11.46), and traditional (cOR 2.40 95% CI 1.05 to 5.47) or Muslim (cOR 5.51 95%CI 1.79 to 16.90) religion vs. non-Apostolic Christians. Compared to those on ART for >2 years, patients who were on ART for <6 months were less likely to have both AD and VLS (cOR 0.41 95% CI 0.26 to 0.65). In contrast, those on ART for 6 months-2 years were more likely to have both AD and VLS (cOR 1.70 95% CI 1.11 to 2.61). Urban versus rural residence, province, wealth quintile, history of CD4 testing and education were not associated with concurrent AD and VLS. In the final, multivariable model adjusting for sex, age, time since ART initiation, and religion, male sex (aOR 2.45 95% CI 1.61 to 3.72), age 35–49 years (aOR 2.46 95%CI 1.03 to 5.91) and 50+ years (aOR 4.82 95% CI 2.02 to 11.46) vs 15–24 years, ART duration (<6 months vs more than 2 years aOR 0.46 95% CI 0.29 to 0.76; 6–24 months vs more than 2 year aOR 2.07 95% CI 1.35 to 3.17), and Muslim religion (aOR 6.36 95%CI 1.70 to 23.76) had significant associations with AD plus VLS. Significant interactions were noted between male sex and the older two age groups. To account for this interaction, we stratified the models on sex. The relationship between sex and AD is driven by age with men aged 25–34, (aOR 3.37 95% CI 1.35 to 8.41), men aged 35–49 years (aOR 5.13 95% CI 2.16 to 12.18), and men aged 50+ (aOR 12.56 95% CI 4.82 to 32.72) all being significantly associated with AD versus men age 15–24. Further, in the male only adjusted model, those who were on ART for <6 months were less likely to have both AD and VLS (cOR 0.29 95% CI 0.14 to 0.62) compared to those on ART for >2 years. In contrast, those on ART for 6 months-2 years were more likely to have both AD and VLS (cOR 2.09 95% CI 1.23 to 3.47).

**Table 3:** Results of univariate and multivariable models illustrating factors associated with advanced HIV disease and concurrent suppressed viral load (n = 167) and further restricted to male sex.

	n	cOR (95% CI)*	aOR (95% CI)**	Where sex = male aOR (95%CI)
**Sex**				
Female	69	Ref.	Ref.	--
Male	98	2.70 (1.73–4.20)	2.45 (1.61–3.72)	--
**Age**				
15–24	65	Ref.	Ref.	Ref.
25–34	76	1.50 (0.58–3.88)	1.68 (0.67–4.23)	3.37 (1.35–8.41)
35–49	21	1.94 (0.72–5.20)	2.46 (1.03–5.91)	5.13 (2.16–12.18)
50+	5	4.16 (1.51–11.46)	4.82 (2.02–11.46)	12.56 (4.82–32.72)
**Residence**				
Urban	51	1.10 (0.74–1.65)	--	
Rural	116	Ref.	--	
**Province**				
Bulawayo	20	Ref.	--	
Manicaland	17	1.41 (0.78–2.56)	--	
Mashonaland Central	12	1.41 (0.77–2.58)	--	
Mashonaland East	19	0.97 (0.55–1.73)	--	
Mashonaland West	11	0.58 (0.29–1.19)	--	
Matebeleland North	12	0.47 (0.21–1.01)	--	
Matebeleland South	16	0.78 (0.42–1.46)	--	
Midlands	22	0.73 (0.37–1.41)	--	
Masvingo	20	0.89 (0.41–1.94)	--	
Harare	18	1.28 (0.67–2.43)	--	
**Wealth Quintile**				
Lowest	26	Ref.	--	
Second	33	0.93 (0.57–1.50)	--	
Third	26	0.65 (0.40–1.07)	--	
Fourth	36	1.00 (0.58–1.72)	--	
Highest	46	0.81 (0.51–1.29)	--	
**Education**				
Primary or below	81	1.24 (0.87–1.76)	--	
Secondary or above	86	Ref.	--	
**CD4 test ever**				
No	140	0.37 (0.24–0.56)	--	
Yes	27	Ref.	--	
**Time since ART initiation**				
<6 months	93	0.41 (0.26–0.65)	0.46 (0.29–0.76)	0.29 (0.14–0.62)
6 months to 2 years	44	1.70 (1.11–2.61)	2.07 (1.35–3.17)	2.09 (1.23–3.47)
More than 2 years	30	Ref.	Ref.	Ref
**Religion**				
Non Apostolic Christian	81	Ref.	Ref.	Ref.
Apostolic	46	0.87 (0.58–1.32)	0.95 (0.61–1.46)	1.20 (0.64–2.52)
Traditional	7	2.40 (1.05–5.47)	1.58 (0.66–3.77)	2.15 (0.81–5.67)
Muslim	4	5.51 (1.79–16.90)	6.36 (1.70–23.76)	10.17 (1.52–68.06)
Other or None	29	1.49 (0.87–2.55)	0.95 (0.61–1.46)	1.52 (0.83–2.77)

*cOR (crude Odds Ratio)

**aOR (adjusted Odds Ratio).

## Discussion

Given the strong correlation between disease progression and HIV viral replication, VL monitoring has replaced CD4 as the recommended standard for laboratory monitoring of ART patients. While some have asserted that immunologic monitoring may no longer be necessary [16], despite significant progress in ART coverage, identifying and caring for PLHIV with AD remains a serious challenge in the region. Our analysis of the ZIMPHIA data reveals a sizeable proportion of PLHIV with AD (17%). This is similar to findings in South Africa, where Carmona and colleagues reported that despite a 30-fold increase in the number of ART patients from 2005–2016, the percentage presenting with AD continues to be over 30% [17]; similar results have been noted in Rwanda, Mozambique, and eSwatini [18,19]. More significantly, we demonstrate that in a high-prevalence setting such as Zimbabwe, a significant number of PLHIV with AD may not be identified if VL testing is used alone, contributing to the growing literature on patients who experience immunosuppression despite ART and virologic response.

In our study, although AD was strongly associated with shorter ART duration (<6 months compared to >2 yrs), multivariable analysis incorporating VLS reversed this association. This is not surprising given that while shorter (or no) ART exposure is strongly associated with AD, the subset of individuals experiencing both AD and VLS are more likely to have had sufficient ART exposure to achieve the latter; nearly 35% of AD patients (irrespective of virologic status) in our analysis reported an ART duration of two or more years. The subset of PLHIV experiencing AD despite prolonged ART and VLS has been noted in several other studies from the region. Nanzigu and colleagues reported a relationship between poor immunologic recovery and AD at the time of ART initiation in Uganda [20], while Asmelash et al noted that suboptimal CD4 recovery despite VLS was associated with older age, hepatitis B virus co-infection, and interestingly, a lower baseline VL [21]. Merci and colleagues in Rwanda reported that after 1 year of ART, among patients with baseline AD, 96% achieved VLS (<20 copies/mL), yet 29% of the cohort still suffered from persistent AD [22]. In a multicenter study in the Democratic Republic of Congo and Kenya, Ousley and colleagues reported that 83–97% of PLHIV were noted to have AD in the inpatient setting; moreover, approximately half of these individuals reported having been on ART for >6 months, and among these, 17–20% were noted to have VLS [23]. The clinical consequences of AD despite VLS have been reported: among PLHIV in South Africa, Takuva and colleagues demonstrated a 2 to 3-fold increase in progression to an AIDS-defining condition, and an increased risk of mortality among patients with AD at 6 months post-ART initiation, despite their having achieved VLS [24]. In the PASER-M cohort representing six countries in sub-Saharan Africa, among patients with VLS, those with poor CD4 recovery (<200) demonstrated significantly higher rates of HIV-related mortality, incidence of AIDS and incidence of pulmonary TB for several years post-ART initiation [25].

The percentage of AD patients who may be missed through VL and clinical monitoring alone (30.1%) and the percentage of virally suppressed patients with AD (8.7%) both highlight the risks of decreased support for CD4 monitoring. Specifically, this implies that thousands of PLHIV (approximately 62,365 in Zimbabwe) may be mistakenly designated “clinically stable,” when in fact they may require additional support. In the REALITY trial, 47% of HIV-infected participants were classified as having WHO clinical stage I and II disease despite having a CD4<100 [26]. As a result, these patients may not receive the WHO recommended package of services to reduce mortality, such as screening for cryptococcal disease and TB lateral flow urine lipoarabinomannan testing for those with CD4 <100 and signs and symptoms of TB [27]. PLHIV with AD may also require prioritization for TB preventive therapy, once active disease has been ruled out.

Zimbabwe’s 2016 ART Outcomes Report noted gender-based differences in immunologic status; median baseline CD4 counts for males and females were 207 and 253, respectively, at the time of ART initiation [28]. We complement that data herein with a nationally representative cross-section of PLHIV stratified by self-reported duration on ART. Unfortunately, males continue to demonstrate poorer immunologic status, evidenced by their over-representation among the AD cohort overall, as well as the subset of AD patients with VLS. Clinicians and public health policy makers may therefore need to strengthen efforts to bolster differentiated care for men (e.g., reach men with treatment literacy messaging to encourage earlier diagnosis, presentation to care, and uptake of the appropriate package of services).

One limitation of this analysis is that ART status and duration on ART are self-reported, which may reflect inaccuracies related to recall as well as participants’ possible reticence in disclosing longstanding HIV-positive status. This is mitigated, however, by the fact that our analysis focuses on the subset of PLHIV with both AD and VLS; the latter serves as a reasonable proxy for true ART adherence. An additional limitation is that this analysis is limited to adults aged 15 and older and as such, our conclusions cannot be extrapolated to include AD among adolescents and children. In light of previous studies revealing 63% of HIV-infected children with AD [29], and persistent delays in ART initiation among children [30] despite the implementation of Treat All policies, further investigation into AD and concurrent VLS among children and adolescents may help guide strategies to improve morbidity and mortality in this population. Our survey results capture cross-sectional data among participants at home; in contrast, longitudinal cohort tracking via case-based surveillance would allow for a more detailed analysis of ART duration and its relationship to AD. In addition, because ZIMPHIA variables did not include clinical staging, we cannot describe the additional effect of clinical staging in identifying PLHIV with AD. An additional limitation of this cross-sectional methodology is that CD4 values may vary significantly over time; participants with AD and <6 months of ART exposure may actually be on an upward trajectory of immune reconstitution. Finally, it should be noted that the proportion of those with both AD and VLS was relatively small within the overall population of PLHIV identified in the study. As such, some of the strata in our analysis contain small unweighted sample sizes.

## Conclusion

As Zimbabwe and other high-burden countries approach epidemic control of HIV through widespread availability and access to ART for millions, it is increasingly important to ensure differentiation of services based upon individual patient circumstances. Given our findings and others in the region, a non-negligible proportion of PLHIV on ART continue to suffer from AD, despite having achieved VLS. Without access to CD4 monitoring, clinical providers may not be aware of their patients’ AD and would therefore be unable to provide the recommended services to prevent avoidable morbidity and mortality. At an aggregate level, the persistence of AD despite VLS may affect the projected impact of ART upon population-level life expectancy. Support for CD4 monitoring, its prioritization among specific sub populations like men, and strengthening the implementation of WHO clinical staging may therefore warrant further consideration. Stronger case-based surveillance with longitudinal, patient-level monitoring in the region would also be useful to quantify the proportion of PLHIV who may suffer long-term AD and allow the public health community to anticipate their needs.

## Supporting information

S1 File (ZIP)
